# Rapid Identification of New Psychoactive Substances in Letters by LA‐APCI‐MS

**DOI:** 10.1002/dta.70003

**Published:** 2025-11-24

**Authors:** Mark Wesner, Hannah Rämisch, Laura Besch, Johannes Schmeinck, Uwe Karst

**Affiliations:** ^1^ Institute of Inorganic and Analytical Chemistry University of Münster Münster Germany; ^2^ State Criminal Police Office of Rhineland‐Palatinate Mainz Germany

**Keywords:** laser ablation, mass spectrometry, new psychoactive substances, prison mail, synthetic cannabinoid receptor agonists

## Abstract

New psychoactive substances (NPS), especially synthetic cannabinoid receptor agonists (SCRA), are increasingly smuggled into prisons via infused mail. Consumption of those substances by inmates in prisons is associated with increased aggression, violence, and organized crime. Onsite detection of infused mail often is challenging. Because the infused papers do not show any visible stains or olfactory changes, physical inspection is often insufficient. The applicability of further conventional on‐site detection methods like immunoassays and sniffer dogs is severely limited. Because of the rapidly changing supply of already circulating and newly emerging NPS, it is impractical to impossible to keep up with the development of immunoassays or the training of sniffer dogs. Hence, confiscated mail samples are routinely tested by either liquid chromatography or gas chromatography coupled to mass spectrometry (MS), which is costly and time‐consuming. In this study, recent advancements in the hyphenation of laser ablation (LA) and molecular MS were investigated regarding the possible application for the rapid and easy detection of NPS in prison mail. Utilizing an in‐house developed LA‐MS hyphenation based on atmospheric pressure chemical ionization (APCI), 31 mail samples confiscated in German prisons were analyzed. It was possible to correctly identify 27 samples containing SCRAs. For these positive samples, it was also possible to detect the specific compounds each paper was infused with. The use of LA‐APCI‐MS has simplified sample preparation and reduced analysis time per sample to 1 min.

## Graphical abstract

Laser ablation coupled to molecular mass spectrometry utilizing a modified atmospheric pressure chemical ionization source was used for the detection of synthetic cannabinoid receptor agonists in infused prison mail samples.

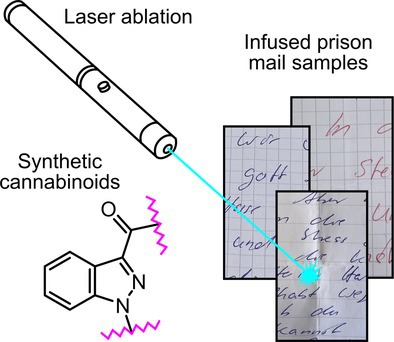

## 1 Introduction

New psychoactive substances (NPS) are a large and diverse group of novel compounds that aim to mimic the psychotropic effects of established drugs of abuse such as cannabis or amphetamines [1, 2, 3]. The number of available NPS is constantly increasing, with 880 compounds being monitored by the former European Monitoring Centre for Drugs and Drug Addiction (EMCDDA) by the end of 2021 [4]. Of those substances, 52 were first reported in 2021, averaging almost one new compound released to the market every week [4, 5]. There are several concerns associated with NPS. The rapid increase of new substances outpaces legal controls and monitoring procedures. This leads to an uncontrolled market of hundreds of novel substances being readily available via online marketplaces, traditional drug dealers and even local shops, so‐called “headshops” [1]. For almost all NPS, there are many unknowns regarding pharmacological and toxicological effects, posing a significant risk for public health [1, 5].

Because NPS are often less expensive despite being much more potent than their traditional analogues, they are especially popular in prisons all around the world [5]. This prevalence is further reinforced with most NPS not being detected during regular urine drug screenings allowing inmates to effectively hide their drug use [1, 2, 3]. According to the EMCDDA, 22 European countries reported NPS use in prisons [6, 7]. Many risks are associated with NPS use in prisons, with increased violence, heightened aggression and organized crime being directly linked to their use [3, 5, 7, 8].

The transition to NPS also changed the routes by which illicit substances enter prisons. While the main smuggling routes previously were via perimeter throwovers, visitors, or corrupt prison staff, infused papers are now the main smuggling route [5, 6, 9, 10]. For the preparation of these papers, NPS are dissolved in an organic solvent. This solution is then either sprayed onto blank paper sheets or the paper itself is dipped directly into the solution [5]. Due to the high potency of most NPS, only small amounts of drug are required for infusion, which results in almost no visible staining or change of the papers' texture [6]. Nevertheless, those papers often contain up to 10% (w/w) of pure NPS [3, 5]. Notably, synthetic cannabinoid receptor agonists (SCRA), which are a subclass of NPS designed to mimic the psychotropic effects of cannabis, are almost exclusively used in this process [6]. The infused papers are then disguised as poems, children's drawings, or regular mail and sent into prison [1, 2]. The inmates then either roll pieces of the infused paper into cigarettes and smoke them similar to cannabis, fit them between the e‐liquid cartridge and heating element of e‐cigarettes, or simply lick or chew the papers [2, 8, 10].

Because there are no obvious stains or olfactory changes to the infused papers, simple physical inspection of incoming prison mail is oftentimes insufficient. Conventional on‐site detection methods like immunoassays or sniffer dogs are also pushed to their limits as it is very time‐consuming and financially demanding to develop new assays or keep training dogs on every new substance released to the pool of already circulating NPS [1, 3]. Seized letters are therefore routinely sent to laboratories where the infused papers are first extracted followed by either gas chromatography (GC) or liquid chromatography (LC) hyphenated to mass spectrometry (MS) [1, 2, 3, 8]. However, this is rather costly and laborious especially regarding the significant number of seized mail [5].

Ion mobility spectrometry (IMS) is sometimes used on‐site for screening of incoming prison mail to reduce the number of suspicious mail samples sent to testing laboratories [5, 6, 10]. Although successful to a certain extent, due to operation by untrained prison staff and the rather low selectivity of IMS for substance mixtures, false positive samples are frequently sent to laboratories, diminishing the overall effect [5, 7]. Hence, analytical methods performed by trained professionals, which are fast and suited for high‐throughput analysis, yet selective, are still needed [7].

Laser ablation (LA) hyphenated to MS presents a method characterized by fast and simple sample introduction. In LA, a pulsed laser beam is focused on the sample, releasing some of it as a sample aerosol, which is then transported to the MS via a carrier gas flow. In contrast to related methods like matrix assisted laser desorption/ionization (MALDI) or secondary ion MS, LA‐MS benefits from operating at ambient pressure as well as a minimum of required sample preparation (e.g., no needed MALDI matrix) [11, 12, 13].

LA is traditionally hyphenated to inductively coupled plasma (ICP)‐MS. In this setup, it is extremely popular for mass spectrometric imaging (MSI), especially in fields like geology, archaeology, biology, and medicine [11, 14]. It is well suited for elemental MSI, but since the sample aerosol is atomized in the ICP prior to the ionization, all molecular information is lost [15]. Hence, there have been several attempts in the past to retain this molecular information by utilizing soft atmospheric pressure ionization approaches. For example, in 2008, Shelley et al. coupled a 266‐nm laser ablation system to MS using flowing atmospheric pressure afterglow ionization [16]. They then utilized that setup for molecular MSI of prints doped with caffeine. Dielectric barrier discharge ionization was later used by Funke et al. for hyphenation of a 213‐nm laser to MS [17]. Here, the setup was used for imaging of coffee beans as well as pain killer tablets. Herdering et al. were the first to hyphenate LA with MS using atmospheric pressure chemical ionization (APCI) [18]. They modified the commercially available APCI source of their orbitrap mass spectrometer using an in‐house built inlet. With that setup, they were able to image the distribution of different drugs on the surface of tablets and thin‐layer chromatography plates. Schmeinck and Karst later used a similar setup for molecular MSI of amino acids in dietary supplement tablets while being able to differentiate between the isomers leucine and iso‐leucine by incorporating an IMS dimension into their setup [15].

In this work, the setup developed by Schmeinck and Karst, which was designed and tested for molecular MSI, is adapted for the fast and easy detection and identification of SCRAs in a large number of infused prison mail samples. In combination with a minimized sample preparation procedure, its applicability as a potential method for high‐throughput analysis of these samples is evaluated.

## 2 Materials and Methods

A total of 31 prison mail samples were provided by the state criminal police office of Rhineland‐Palatinate. Using a hole puncher, a circular cutout with a diameter of roughly 4 mm was created for every sample and fixed to a microscopy slide afterward using double‐sided tape.

**FIGURE 1:**
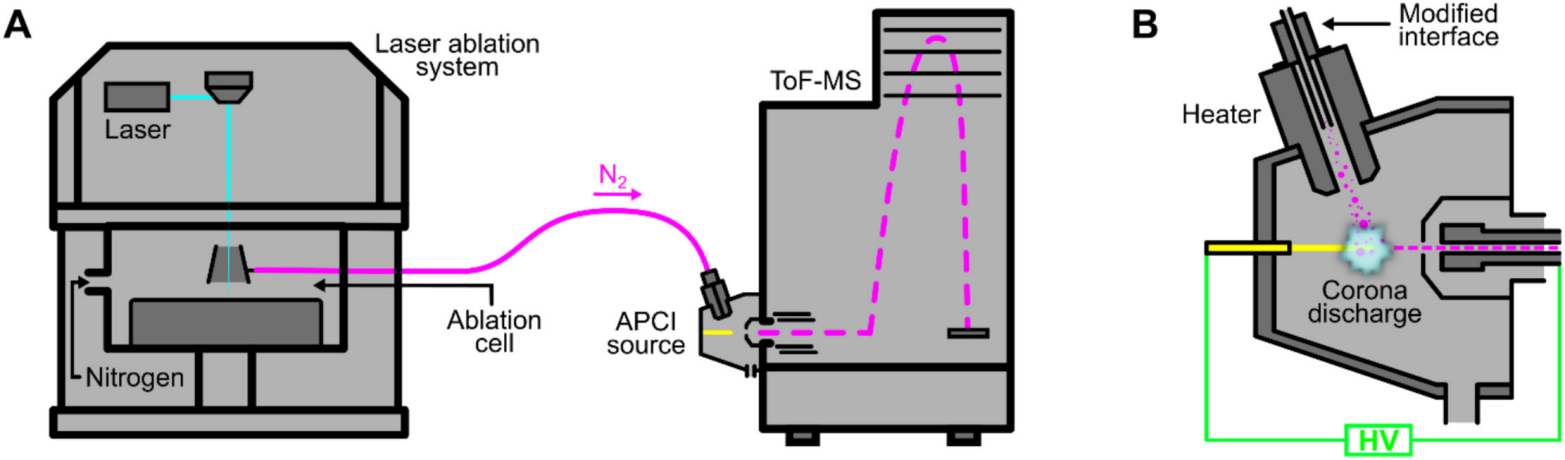
Schematic overview of the hyphenation of a laser ablation system and a mass spectrometer using APCI (A) and the modified APCI source used (B). Based on Schmeinck and Karst [15].

Subsequent analysis of prison mail samples was performed by LA‐APCI‐MS. Figure 1 depicts an overview of the employed setup. An imageBIO266 system (Elemental Scientific Lasers, Bozeman, MT, USA) equipped with a TwoVol2 ablation chamber and a frequency‐quadrupled 266‐nm diode‐pumped Nd:YAG laser was used for LA. For each sample, a 3‐mm‐long line ablation was performed at a laser repetition rate of 10 Hz with a rectangular spot shape, a spot size of 50 μm, a laser fluence of 1 J cm^−1^, and a scan speed of the sample stage of 50 μm s^−1^. For mass spectrometric detection, the generated sample aerosol was transported to a micrOTOF time‐of‐flight (ToF)‐MS (Bruker Daltonics, Bremen, Germany) by a constant nitrogen carrier gas flow of 0.8 L min^−1^. The MS was controlled by micrOTOFcontrol 3.0 and was equipped with an LC‐APCI II ion source (both Bruker Daltonics, Bremen, Germany). The APCI source was modified in‐house to allow the introduction of a dry sample aerosol instead of liquid samples. Further information on the necessary modifications to the ion source is provided in the study by Schmeinck and Karst [15]. The source's built‐in heater was set to a temperature of 250°C and the corona discharge current to 3.5 μA. For data acquisition, the MS was set to a spectra rate of 1 Hz. Detailed mass spectrometric parameters are provided in the Supporting Information (Section S1, Table S1.1). Data evaluation was performed using Compass DataAnalysis 6.1 (Bruker Daltonics, Bremen, Germany) where all acquired spectra per line ablation were averaged.

## 3 Results and Discussion

Prison mail samples, suspected to be infused with NPS, were studied by means of LA‐APCI‐MS. The used instrumental setup was developed by Schmeinck and Karst [15]. In their study, they modified a commercially available APCI source to allow the ionization of a dry sample aerosol generated by LA instead of liquid samples and used it for molecular MSI. In MSI, the laser beam is focused on multiple spots across a single sample, sequentially scanning the whole sample in order to obtain spatial resolution. In the present study, this exact setup is also used, although not because of its imaging capabilities, but rather because of the minimal sample preparation needed and the high‐throughput analysis it allows. Because the system was optimized for fast imaging of single samples, rapid analysis of multiple samples can be achieved by focusing the laser beam on only one spot per sample across a set of multiple samples.

Traditionally, LC‐ or GC‐MS methods are used to test suspicious prison mail for NPS, which require laborious extraction steps and are generally time‐consuming. Here, chromatographic separation alone typically takes 10–20 min per sample [1, 2, 3, 8]. Using LA‐APCI‐MS, sample preparation was reduced to sampling each letter using a hole puncher and fixing the resulting cutouts to microscopy slides using double‐sided tape. Because the laser beam only penetrates the sample surface in the low micrometer range during LA and because the NPS are primarily located on the sample surface, mainly NPS are transferred to the resulting sample aerosol. This allows a direct analysis of mail samples in contrast to common liquid extraction, where major parts of a sample's constituents are extracted, making chromatographic separation mandatory.

**FIGURE 2:**
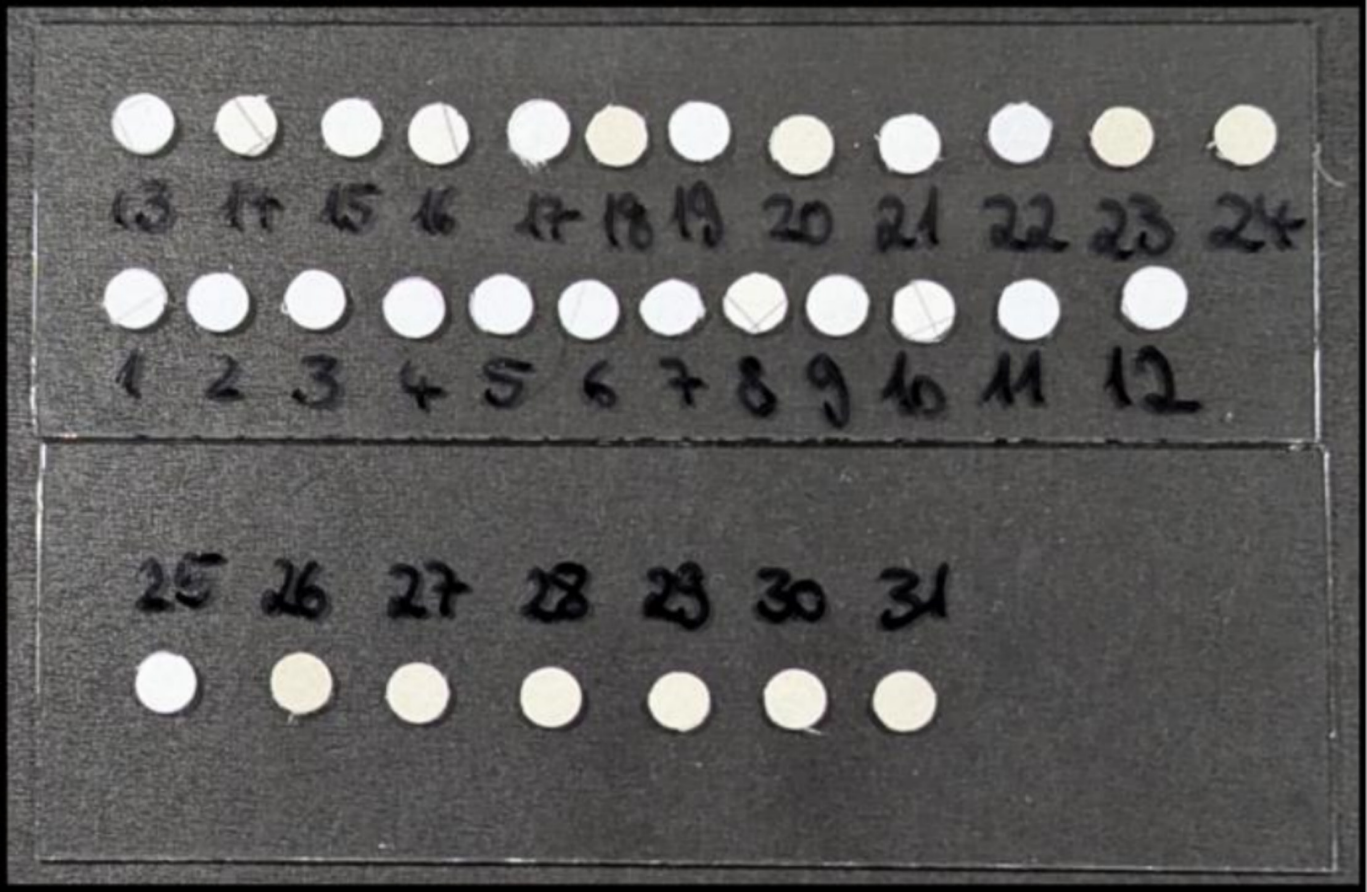
Image of all 31 mail sample cutouts mounted onto two microscopy slides.

Figure 2 shows a photograph of all 31 investigated samples being mounted onto two microscopy slides. Before providing the samples, GC‐MS analysis was carried out by the state criminal police office of Rhineland‐Palatinate to determine whether the letters were in fact infused with NPS and if so, which exact compounds were present. To ensure an unbiased approach, these results were not shared with us prior to our analysis, with the exception of one sample, which was selected randomly to facilitate method development. Therefore, the analyses were performed as a blinded study.

**FIGURE 3:**
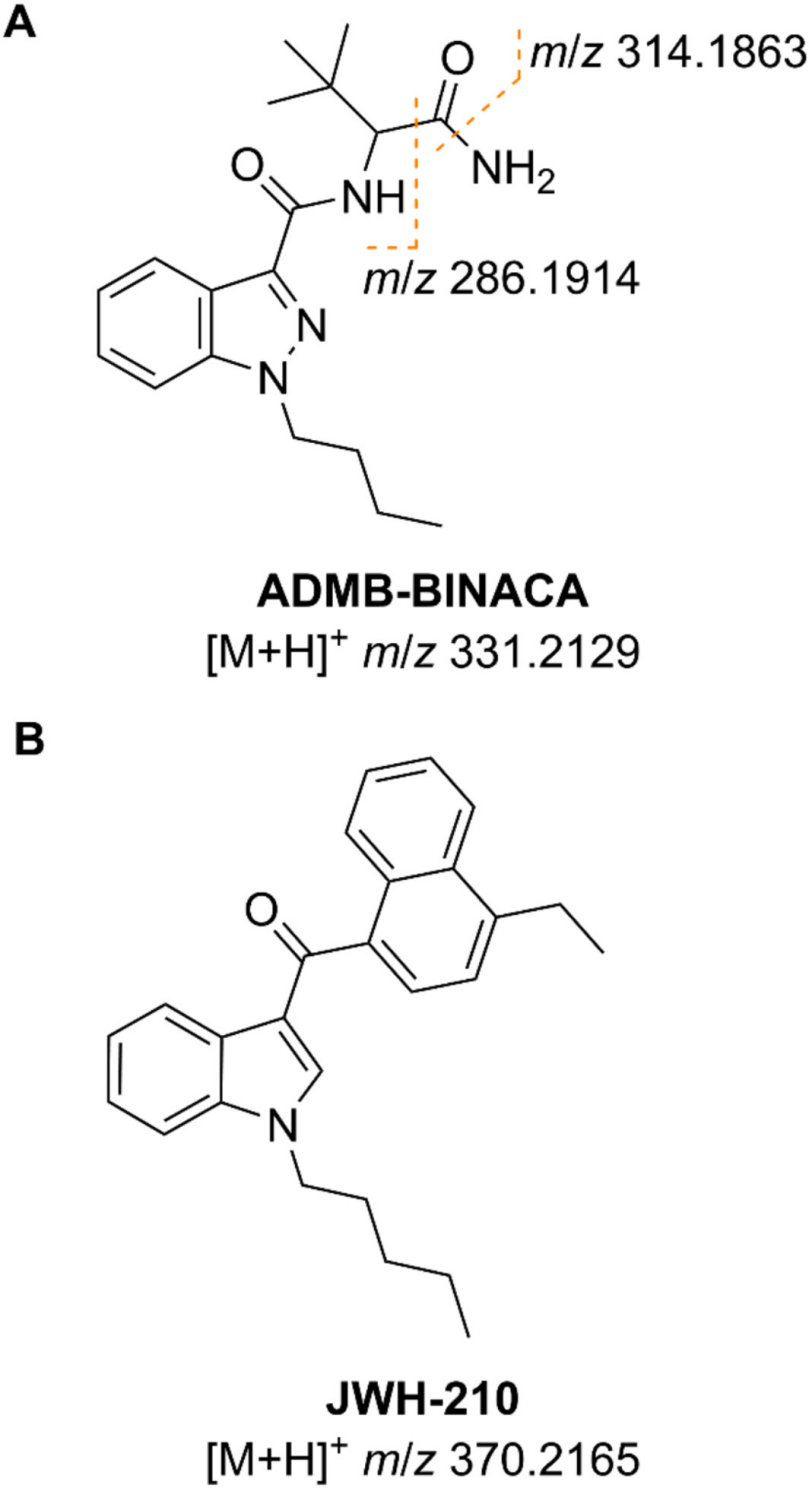
Molecular structure of SCRAs. ADMB‐BINACA with proposed fragmentation pattern (A) and JWH‐210 (B).

The randomly selected sample is labeled **18** in Figure 2. It contained two distinct substances, namely ADMB‐BINACA (*N*‐[(2*S*)‐1‐amino‐3,3‐dimethyl‐1‐oxobutan‐2‐yl]‐1‐butylindazole‐3‐carboxamide, Figure 3A) and JWH‐210 ((4‐ethylnaphthalen‐1‐yl)‐(1‐pentylindol‐3‐yl)methanone, Figure 3B). ADMB‐BINACA is a potent full agonist of both cannabinoid type 1 (CB_1_) and type 2 (CB_2_) receptors. It causes strong psychotropic effects similar to those caused by the partial CB_1_ and CB_2_ receptor agonist Δ^9^‐tetrahydrocannabinol (Δ^9^‐THC), the main active ingredient in natural cannabis [19, 20]. Unlike cannabis, ADMB‐BINACA is also known to cause many adverse side effects, for example severe hepatotoxicity likely caused by induction of oxidative stress [21]. ADMB‐BINACA was first detected by government authorities in Sweden back in 2019 [19, 22]. JWH‐210, on the other hand, was first synthesized by the research group of J.W. Huffman in 2005 as a tool to study the human endocannabinoid system and cannabinoid receptors, but was then also found in illicit products obtained via the internet in 2011 in Japan [23, 24]. Similar to ADMB‐BINACA, JWH‐210 is also a full agonist of the CB_1_ receptor as well as a potent partial agonist of the CB_2_ receptor. It is known to cause many adverse side effects with tachycardia, nausea and hypertension being reported most frequently [25].

**FIGURE 4:**
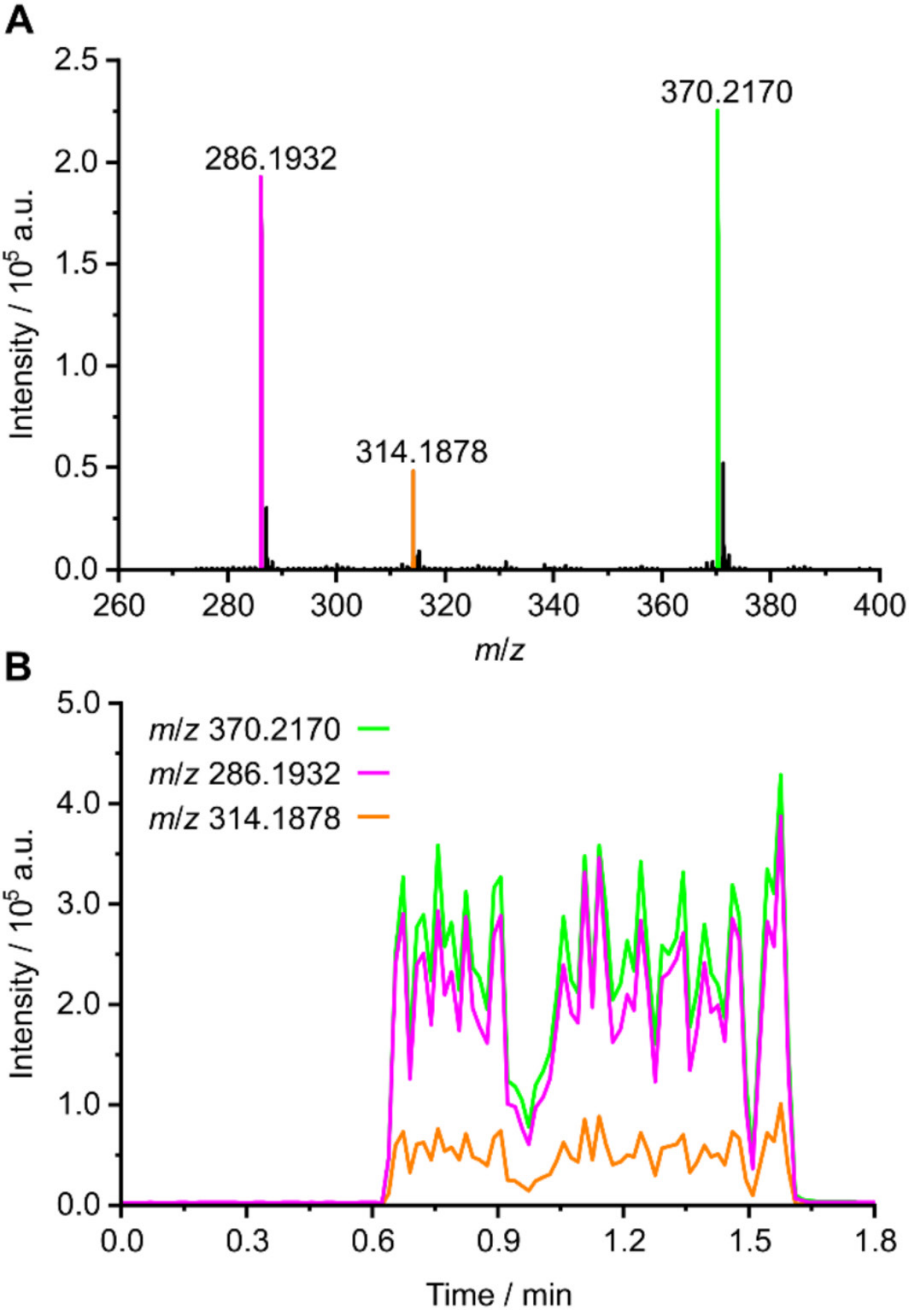
Average mass spectrum obtained by LA‐APCI‐MS (A) as well as EICs for the observed signals (B) for prison mail sample **18**.

Figure 4A shows the mass spectrum obtained by LA‐APCI‐MS for the above‐mentioned mail sample. It was acquired by ablating a line over the sample for 1 min and averaging the recorded mass spectra in data processing afterwards. Parameters for both LA and MS were optimized to yield maximized signal intensities with minimal fragmentation. Three distinct signals can be observed at different mass‐to‐charge ratios (*m*/*z*), namely, *m*/*z* 286.1932, *m*/*z* 314.1878, and *m*/*z* 370.2170. Figure 4B shows the extracted ion currents (EIC) for all three signals. Line ablation started at around 0.6 min and lasted for 1 min. It can be observed that the EICs for all three signals greatly increase during laser ablation and decrease again afterwards when the laser is turned off. This indicates that the signals in fact originate from substances that are removed from the mail sample during laser ablation. Furthermore, it is observed that the EICs for all three signals greatly fluctuate during the continuous laser ablation, suggesting a heterogenous distribution of NPS across the mail sample. Here, single spot ablation could result in the analysis of only a sample region containing no to minimal analyte while line ablation samples, a greater surface area assures the analysis of sample regions containing large amounts of analyte. Hence, line ablation was selected for analysis of all following samples.

The signal at *m*/*z* 370.2170 corresponds to the protonated molecular ion of JWH‐210, which would be observed at an exact *m*/*z* of 370.2165. The remaining two signals correspond to fragments originating from ADMB‐BINACA. The ion at *m*/*z* 314.1878 is most likely formed by cleavage of the C–N bond of its terminal amide group resulting in an exact *m*/*z* of 314.1863. The ion at *m*/*z* 286.1932 is probably formed by further loss of carbon monoxide resulting in an exact *m*/*z* of 286.1914. Since both fragmentation pathways are not commonly observed in traditional APCI or electrospray ionization modes, it can be proposed that fragmentation does not occur during the ionization process but rather during laser ablation. The exposure of the sample to the high‐energy laser beam likely induces cleavage of the analytes resulting in the fragments observed in Figure 4. The proposed fragmentation pattern is shown in Figure 3A. Accurate *m*/*z* together with proposed ion formulae, exact *m*/*z* and mass deviations are summarized in Table 1.

**TABLE 1:** Accurate *m*/*z*, proposed ion formulae, exact *m*/*z*, and mass deviations for all signals observed during LA‐APCI‐MS analysis of prison mail sample **18**.

Compound	Accurate *m*/*z*	Ion formula	Exact *m*/*z*	Δ*m*/*z*/ppm
JWH‐210	370.2170	C_26_H_28_NO^+^	370.2165	−2.1
ADMB‐BINACA F1	314.1878	C_18_H_24_N_3_O_2_^+^	314.1863	−4.8
ADMB‐BINACA F2	286.1932	C_17_H_24_N_3_O^+^	286.1914	−6.3

*Note:* Compound “ADMB‐BINACA F1” refers to the fragment of ADMB‐BINACA formed likely by cleavage of the C‐N‐bond of the terminal amide group, while “ADMB‐BINACA F2” refers to the fragment probably formed by further loss of carbon monoxide.

Following method optimization, all remaining samples were analyzed by this LA‐APCI‐MS approach. Each sample was ablated for 1 min, leading to a total analysis time of just over 30 min for all 31 samples, demonstrating a stark contrast to the 5 to 10 h traditional LC–MS or GC‐MS analysis would take. Signals for both JWH‐210 and ADMB‐BINACA were observed in 9 further samples resulting in a total of 10 samples that were presumably infused with a mixture of both substances. In 15 of the remaining 21 samples, only the two fragment signals originating from ADMB‐BINACA were observed, implying that those samples were infused with only this one SCRA. No signals that differ from the background signals, which are observed when the laser ablation system was turned off, were detected for a total of 4 samples. These samples were therefore regarded as negative samples. It could be possible for regular prison mail to be confiscated and sent in for analysis if it looked suspicious, for example by featuring visible stains that are not originating from illicit substances. Finally, even though signals were observed for the two remaining samples, they neither matched the signals observed for JWH‐210 nor those for ADMB‐BINACA. In Figure 2, these samples are labeled **14** and **16** respectively.

**FIGURE 5:**
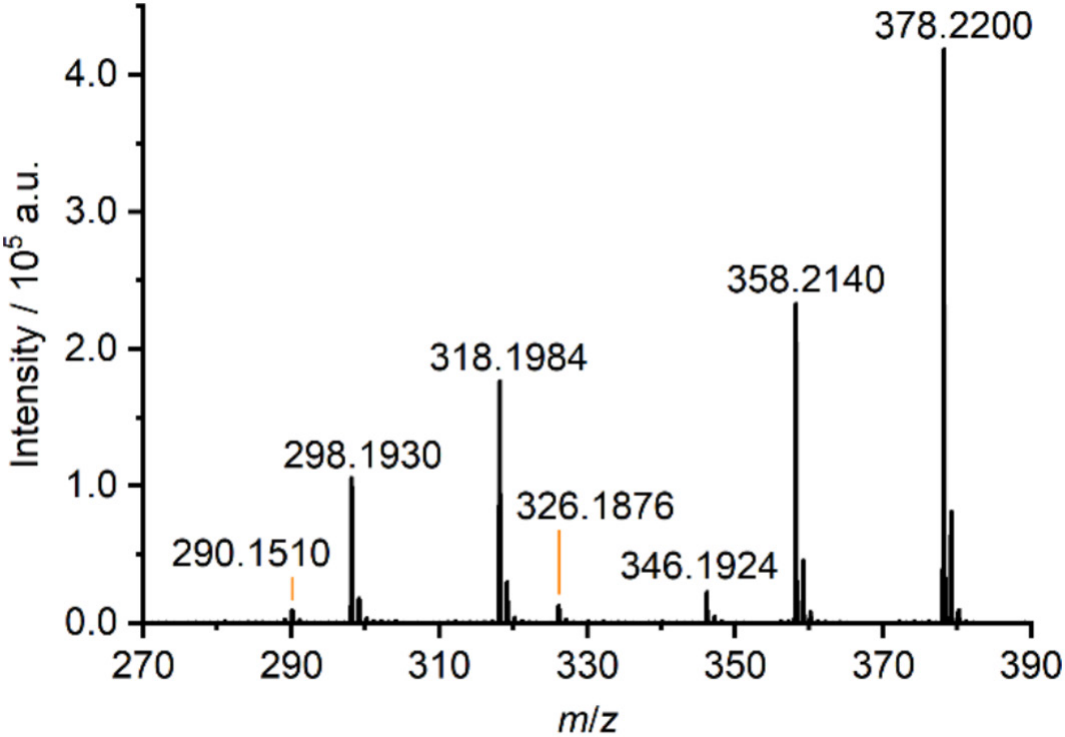
Average mass spectrum obtained by LA‐APCI‐MS for prison mail sample **16**.

Figure 5 shows the mass spectrum obtained by LA‐APCI‐MS for prison mail sample **16**. Because the obtained mass spectra for samples **14** and **16** are almost identical, only one mass spectrum is shown here exemplarily. Seven distinct signals can be observed in this mass spectrum. Because the compounds corresponding to the signals were unknown this time, the signals were matched against the NPS Data Hub database. This database is a web‐based data repository for analytical data specifically related to NPS [26]. Using this approach, the signals at *m*/*z* 378.2200, *m*/*z* 358.2140, and *m*/*z* 290.1510 were matched to the protonated molecular ions of three distinct SCRAs.

**FIGURE 6:**
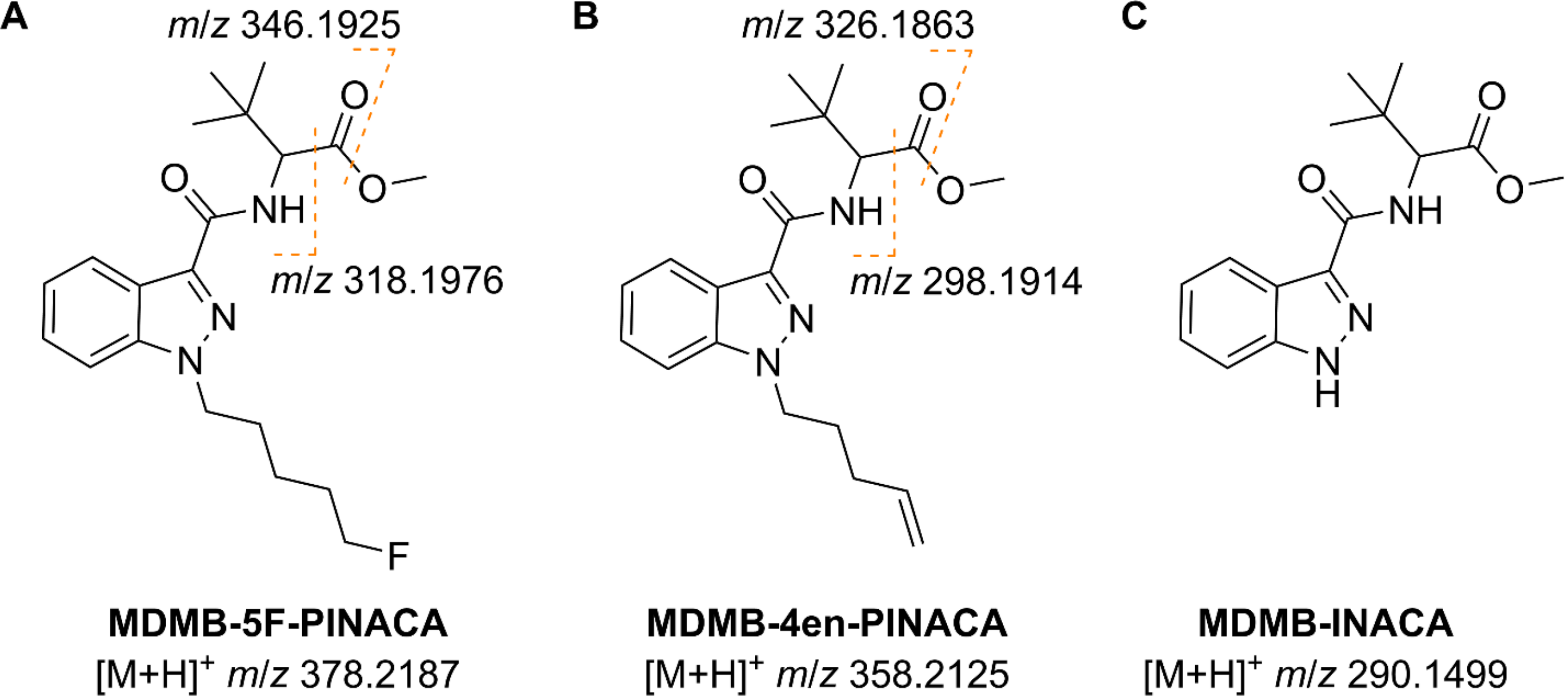
Molecular structures of the SCRAs MDMB‐5F‐PINACA (A), MDMB‐4en‐PINACA (B), and MDMB‐INACA (C) with proposed fragmentation patterns.

The most intense signal in the spectrum at *m*/*z* 378.2200 corresponds to the protonated molecular ion for MDMB‐5F‐PINACA (methyl (S)‐2‐[1‐(5‐fluoropentyl)‐1H‐indazole‐3‐carboxamido]‐3,3‐dimethylbutanoate, Figure 6A), which would be observed at an exact *m*/*z* of 378.2187. Similar to the other SCRAs mentioned above, MDMB‐5F‐PINACA is also a potent agonist of the CB_1_ receptor and therefore causes the cannabis‐like effects expected by most users [27, 28]. It also causes unexpected and unwanted adverse side effects like tachycardia, psychomotor agitation, confusion and anxiety [29]. MDMB‐5F‐PINACA was first identified in white powder seized in Hungary in 2014 [28]. Based on this database match, the signals at *m*/*z* 346.1924 and *m*/*z* 318.1984 can be explained as fragments originating from MDMB‐5F‐PINACA. The ion at *m*/*z* 346.1924 is most likely formed by loss of methanol from the methyl butanoate moiety, which would result in an exact *m*/*z* of 346.1925, while the ion at *m*/*z* 318.1984 is probably formed by loss of the whole methyl ester group altogether, resulting in an exact *m*/*z* of 318.1976. The proposed fragmentation pattern is shown in Figure 6A.

Furthermore, MDMB‐4en‐PINACA (methyl (S)‐3,3‐dimethyl‐2‐(1‐(pent‐4‐en‐1‐yl)‐1H‐indazole‐3‐carboxamido)butanoate, Figure 6B) was matched to the second most intense signal of the spectrum at *m*/*z* 358.2140, which corresponds to the expected *m*/*z* of 358.2125 for its protonated molecular ion. MDMB‐4en‐PINACA was first identified in seized material in Germany in 2017 [30]. Its molecular structure only differs slightly from MDMB‐5F‐PINACA, which features a 5‐fluoropentyl tail group while MDMB‐4en‐PINACA features a 4‐pentene tail group. It also is a potent full agonist of the CB_1_ receptor [27, 31]. Thus, it is regularly consumed as a substitute for regular cannabis although many users self‐report adverse side effects like chest pains, irregular heartbeat, vomiting, confusion and auditory as well as visual hallucinations [30, 31]. The observed signals at *m*/*z* 326.1876 and *m*/*z* 298.1930 indicate that MDMB‐4en‐PINACA shows the same fragmentation pattern as MDMB‐5F‐PINACA. Here, the ion at *m*/*z* 326.1876 is probably the product from the loss of methanol while the ion at *m*/*z* 298.1930 is most likely formed by the loss of the whole methyl ester which theoretically would be observed at *m*/*z* 326.1863 and *m*/*z* 298.1914, respectively. The proposed fragmentation pattern is shown in Figure 6B.

Finally, the signal at *m*/*z* 290.1510 was assigned to MDMB‐INACA (methyl (S)‐2‐(1H‐indazole‐3‐carboxamido)‐3,3‐dimethylbutanoate, Figure 6C). Here, the protonated molecular ion would theoretically be observed at an exact *m*/*z* of 290.1499. In contrast to other SCRAs, MDMB‐INACA is a rather weak agonist of the CB_1_ receptor and therefore causes less potent cannabis‐like effects. It is rather considered a precursor sold in “Do‐It‐Yourself (DIY)” kits for the synthesis of controlled SCRAs [32]. These kits became common after China, the suspected origin of most internationally sold SCRAs, introduced a class‐wide ban on SCRAs in 2021 [8, 9, 33]. Since then, MDMB‐INACA has been detected in seized material in many different European countries [32, 34]. Although MDMB‐INACA features the same methyl‐3,3‐dimethylbutanoate moiety for the linked group as MDMB‐5F‐PINACA and MDMB‐4en‐PINACA, no fragment ions were observed for MDMB‐INACA. This might be explained through the rather low abundance of MDMB‐INACA in the tested mail samples, where possible fragments are either below the detection limit or are affected by ion suppression effects. Accurate *m*/*z* together with proposed ion formulae, exact *m*/*z* and mass deviations are summarized in Table 2 for all seven signals observed in the two mail samples.

**TABLE 2:** Accurate *m*/*z*, proposed ion formulae, exact *m*/*z*, and mass deviations for all signals observed during LA‐APCI‐MS analysis of prison mail sample **16**.

Compound	Accurate *m*/*z*	Ion formula	Exact *m*/*z*	Δ*m*/*z*/ppm
MDMB‐5F‐PINACA	378.2200	C_20_H_29_FN_3_O_3_^+^	378.2187	−3.7
MDMB‐5F‐PINACA F1	346.1924	C_19_H_25_FN_3_O_2_^+^	346.1925	0.4
MDMB‐5F‐PINACA F2	318.1984	C_18_H_25_FN_3_O^+^	318.1976	−2.5
MDMB‐4en‐PINACA	358.2140	C_20_H_28_N_3_O_3_^+^	358.2125	−4.1
MDMB‐4en‐PINACA F1	326.1876	C_19_H_24_N_3_O_2_^+^	326.1863	−4.0
MDMB‐4en‐PINACA F2	298.1930	C_18_H_24_N_3_O^+^	298.1914	−5.4
MDMB‐INACA	290.1510	C_15_H_20_N_3_O_3_^+^	290.1499	−3.7

*Note:* The suffix “F1” refers to the fragments most likely formed by loss of methanol from the methyl butanoate moiety, while the suffix “F2” refers to the fragments likely formed by loss of the whole methyl ester group.

Following analysis of all 31 prison mail samples, the results were compared to those obtained by GC‐MS by the state criminal police office of Rhineland‐Palatinate for method validation. Comparison showed that all 27 samples infused with SCRAs were identified correctly by LA‐APCI‐MS as positive samples and that there were in fact four negative samples where no SCRAs were detected. Furthermore, identification of the specific SCRAs present in the positive samples matched fully with 10 samples containing a mixture of ADMB‐BINACA and JWH‐210, 15 samples being infused with solely ADMB‐BINACA and the two remaining samples being laced with MDMB‐5F‐PINACA, MDMB‐4en‐PINACA and MDMB‐INACA. A detailed overview of the qualitative results is provided in the Supporting Information (Section S2, Table S2.1).

## 4 Conclusion

In this study, LA‐APCI‐MS was successfully applied for the fast and easy identification of NPS, specifically SCRAs, in confiscated prison mail samples expected to have been infused with illicit substances. A total of 31 samples, confiscated in German prisons and supplied by the state criminal police office of Rhineland‐Palatinate, were tested in a blinded fashion. From those samples, 27 samples were correctly identified as being infused with SCRAs as verified by the state criminal police office by GC‐MS analysis. The remaining four samples were correctly identified as regular blank prison mail. Furthermore, it was possible to identify the specific compounds present in each of the 27 positive samples even for samples infused with up to three different SCRAs.

Using LA‐APCI‐MS reduced the required sample preparation to a minimum. No extraction or derivatization steps were required and samples just needed to be cut to size and fixed to microscopy slides. Analysis times were also drastically reduced from typically 10–20 min per sample to just 1 min per sample. A further reduction of analysis time could be achieved by shortening the ablated lines. However, due to the heterogeneous distribution of SCRAs on the infused papers, this could lead to false negative results.

For almost all analytes, intact protonated molecular ions were observed with additional fragment ions being observed for several analytes. It was not investigated whether fragmentation is taking place during the laser ablation process or as in‐source fragmentation during APCI. Yet, compound identification benefited from the analyte‐specific fragmentation patterns and identification confidence was greatly enhanced in contrast to compound identification solely based on the accurate *m*/*z* of the intact protonated molecular ions. In the future, the addition of these fragmentation patterns to NPS specific databases such as the NPS Data Hub could facilitate the identification of SCRAs by LA‐APCI‐MS even more. Since the presented setup utilized high‐resolution MS, recording accurate *m*/*z* values for both intact molecular ions as well as fragments, matching these analyte‐specific fragmentation patterns would lead to high confidence compound identification. Therefore, further confirmation by established methods like GC‐MS, which typically still rely on unit‐resolution MS, would not be required.

Overall, LA‐APCI‐MS is a new and powerful method for high‐throughput analysis of confiscated prison mail, the need of which was pointed out in the literature before [7]. In contrast to similar methods like MALDI‐MS, for example, LA‐APCI‐MS additionally benefits from the possibility to hyphenate LA to almost all commercially available molecular mass spectrometers and APCI sources. Therefore, laboratories could minimize upfront costs by equipping existing mass spectrometers with laser ablation systems, thus making a widespread adaptation practicable.

## Conflicts of Interest

The authors declare no conflicts of interest.

## Supporting information


**Supporting Information S1:** Supporting information.

## Data Availability

The data that support the findings of this study are available from the corresponding author upon reasonable request.
