# Supplementary material for: Rapid Identification of New Psychoactive Substances in Letters by LA‐APCI‐MS
Source: Drug Test Anal. 2025 Nov 24;18(2):198–206. doi: 10.1002/dta.70003 (PMC12861598; doi:10.1002/dta.70003)
Supplement: Supplementary file 1 — Supporting Information S1: Supporting information. [file DTA-18-198-s001.docx]

**Supporting Information**

**Rapid Identification of New Psychoactive Substances in Letters by LA-APCI-MS** Mark Wesner^a^, Hannah Rämisch^a^, Laura Besch^b^, Johannes Schmeinck^a^ and Uwe Karst^a^*

^a^ Institute of Inorganic and Analytical Chemistry, University of Münster, Corrensstraße 48, 48149 Münster, Germany

^b^ State Criminal Police Office of Rhineland-Palatinate, Valenciaplatz 7, 55118 Mainz, Germany

* Corresponding author: uk@uni-muenster.de

# SI-1 Additional experimental details

Exact mass spectrometric parameters used for the detection of synthetic cannabinoid receptor agonists in prison mail samples by laser ablation (LA) hyphenated to mass spectrometry (MS) using atmospheric pressure chemical ionization (APCI) are listed in Table SI-1.1.

Table SI-1.1: Mass spectrometric parameters used for LA-APCI-MS analysis of infused prison mail samples.

| **Parameter** | **Description** |
| --- | --- |
| **Ionization mode** | Positive ion APCI |
| **Spectra Rate** | 1 Hz |
| **Source** |  |
| **End Plate Offset** | -500 V |
| **Capillary** | -1500 V |
| **Corona** | +3500 nA |
| **Nebulizer** | 0 bar |
| **Dry Gas** | 4 l min^-1^ |
| **Dry Temp** | 200 °C |
| **Vaporizer Temp** | 250 °C |
| **Transfer** |  |
| **Capillary Exit** | 150 V |
| **Skimmer 1** | 50 V |
| **Hexapole 1** | 23.1 V |
| **Hexapole RF** | 120 V_pp_ |
| **Skimmer 2** | 23.1 V |
| **Lens 1 Transfer** | 45 µs |
| **Lens 1 Pre Puls Storage** | 10 µs |

# SI-2 Detailed experimental results

Experimental results for the qualitative analysis of all 31 infused prison mail samples by LA-APCI-MS as well as gas chromatography (GC)-MS are shown in Table SI-2.1. When provided, quantitative results are listed additionally. Quantitative analysis was carried out by the state criminal police office of Rhineland-Palatinate by liquid chromatography (LC) with diode array detection (DAD).

Table SI-2.1: Qualitative results for the analysis of infused prison mail samples by LA-APCI-MS and GC-MS as well as qualitative results for several samples obtained by LC-DAD.

| **Sample** | **LA-APCI-MS result** | **GC-MS result** |
| --- | --- | --- |
| **1** | ADMB-BINACA | ADMB-BINACA |
| **2** | ADMB-BINACA | ADMB-BINACA (4.18%*w*/*w*) |
| **3** | ADMB-BINACA | ADMB-BINACA |
| **4** | ADMB-BINACA | ADMB-BINACA |
| **5** | ADMB-BINACA | ADMB-BINACA (4.13%*w*/*w*) |
| **6** | ADMB-BINACA | ADMB-BINACA (4.31%*w*/*w*) |
| **7** | ADMB-BINACA | ADMB-BINACA (3.98%*w*/*w*) |
| **8** | negative | Negative |
| **9** | negative | Negative |
| **10** | negative | Negative |
| **11** | ADMB-BINACA | ADMB-BINACA (4.09%*w*/*w*) |
| **12** | negative | Negative |
| **13** | ADMB-BINACA | ADMB-BINACA |
| **14** | MDMB-4en-PINACA, MDMB-5F-PINACA, MDMB-INACA | MDMB-4en-PINACA (1.43%*w*/*w*),  MDMB-5F-PINACA, MDMB-INACA |
| **15** | ADMB-BINACA | ADMB-BINACA (4.79%*w*/*w*) |
| **16** | MDMB-4en-PINACA, MDMB-5F-PINACA, MDMB-INACA | MDMB-4en-PINACA (1.59%*w*/*w*),  MDMB-5F-PINACA, MDMB-INACA |
| **17** | ADMB-BINACA | ADMB-BINACA (5.40%*w*/*w*) |
| **18** | ADMB-BINACA, JWH-210 | ADMB-BINACA (3.35%*w*/*w*), JWH-210 |
| **19** | ADMB-BINACA | ADMB-BINACA (4.85%*w*/*w*) |
| **20** | ADMB-BINACA, JWH-210 | ADMB-BINACA (2.96%*w*/*w*), JWH-210 |
| **21** | ADMB-BINACA | ADMB-BINACA (5.79%*w*/*w*) |
| **22** | ADMB-BINACA | ADMB-BINACA (5.90%*w*/*w*) |
| **23** | ADMB-BINACA, JWH-210 | ADMB-BINACA (4.73%*w*/*w*), JWH-210 |
| **24** | ADMB-BINACA, JWH-210 | ADMB-BINACA (3.84%*w*/*w*), JWH-210 |
| **25** | ADMB-BINACA | ADMB-BINACA (4,40%*w*/*w*) |
| **26** | ADMB-BINACA, JWH-210 | ADMB-BINACA (4.11%*w*/*w*), JWH-210 |
| **27** | ADMB-BINACA, JWH-210 | ADMB-BINACA (3.47%*w*/*w*), JWH-210 |
| **28** | ADMB-BINACA, JWH-210 | ADMB-BINACA (2.87%*w*/*w*), JWH-210 |
| **29** | ADMB-BINACA, JWH-210 | ADMB-BINACA (3.91%*w*/*w*), JWH-210 |
| **30** | ADMB-BINACA, JWH-210 | ADMB-BINACA (3.75%*w*/*w*), JWH-210 |
| **31** | ADMB-BINACA, JWH-210 | ADMB-BINACA (4.44%*w*/*w*), JWH-210 |
